# Frequency of C-peptide and antibody levels (anti GAD, Islet cell antibodies, insulin auto antibodies) in children of Pakistan with Type-1 diabetes

**DOI:** 10.12669/pjms.40.6.7794

**Published:** 2024-07

**Authors:** Maira Riaz, Marium Akram, Mohsina Noor Ibrahim, Zubair Ahmed Khoso

**Affiliations:** 1 Maira Riaz, FCPS, MBBS. Assistant Professor, National Institute of Child Health, Karachi, Pakistan; 2 Marium Akram, MBBS. Registrar, National Institute of Child Health, Karachi, Pakistan; 3 Mohsina Noor Ibrahim, FCPS, DCH, MCPS, MBBS Professor, National Institute of Child Health, Karachi, Pakistan; 4 Zubair Ahmed Khoso, FCPS, MBBS, Assistant Professor, National Institute of Child Health, Karachi, Pakistan

**Keywords:** Type-1 DM, Insulin autoantibodies, C-peptide, Anti GAD antibodies

## Abstract

**Background and Objective:**

The autoimmune mechanism in T1DM causes gradual loss of pancreatic β-cell, which progresses to hyperglycemia and ultimate reliance on consistent insulin therapy. T1DM has been the commonest type of diabetes in children and this study will help in refining indulgent towards the problem and its pathophysiology in our people. The objective was to find out the prevalence of C-peptide and antibody levels (anti GAD, ICA, IAA and IA2) in children and adolescents of Pakistan with T1DM.

**Methods:**

We conducted this cross-sectional study at Department of Pediatric Endocrinology, National Institute of Child Health, Karachi between August 2019 to February 2020 and included 98 children who had T1DM for more than one month. Subjects whose GFR was <30ml/min were omitted from the study. Among those registered subjects, C-peptide, human islet cell antibody (ICA), insulin auto antibodies (IAA) and anti-glutamic acid decarboxylase were assessed. Demographical and laboratorial facts were noted on a pre-constructed proforma.

**Results:**

There were 77(78.3%) cases who had level of C-peptide <0.8 and anti-GAD was found in 47(48%) subjects. 35(35.7%) cases found positive for IA2 .and 7(7.1%) patients had insulin auto antibodies positive while ICA was negative in total 98(100%) subjects.

**Conclusion:**

Children with T1 DM possessed increased levels of anti-GAD antibodies, insulin autoantibodies and anti (IA2) but islet cells antibodies were negligible in our population when checked at a point of time. C-peptide may be normal in some, but its level declines with long duration of diabetes in children.

## INTRODUCTION

Type-1 diabetes, an autoimmune disease is said to be the commonest type of diabetes in children, and its incidence is on the rise.^1^ It is predicted that in under five years European children, it will increase two-fold in less than 20 years. According to one meta-analysis, the frequency of T1DM in Asia, Africa, Europe, and America was 15 per 100,000, eight per 100,000, 15 per 100,000 and 20 per 100, respectively. While its worldwide prevalence in these regions was, 6.9 per 10,000, 3.5 per 10,000, and 12.2 per 10,000, respectively.^2^ Though males more commonly present with this illness in initial adult life, the incidence of T1DM is equivalent in both genders during childhood.^3^ Onset of T1DM usually depends on environmental triggers, recruitment and activation of immune cells and genetic predisposition.^4^

Type-1 diabetics are unable to produce insulin, therefore level of C-terminal cleavage product (C-peptide) produced during processing of the insulin pro-hormone to the mature insulin molecule is found low in them.^5^ Usually, in T1DM, C-peptide secretion decreases with time and the rate of decline is faster in younger age of onset.^6^ Data from Pakistan suggests that C-peptide levels maintains itself in young people with T1DM.^7^ C-peptide levels differ in various populations at the time of diagnosis but generally it correlates with type of diabetes, duration of disease, and age at the time of diagnosis. Multiple islet autoantibodies are found in the large majority of children who develop Type-1 diabetes, Type-1 diabetes in adulthood often present with only GAD antibodies.^5^ As per western literature, the prevalence of GAD autoantibodies, IA-2 autoantibodies, and ZnT8 autoantibodies are 70%-80%, 60%-70%, and 60%-80%, respectively in children with new onset T1DM.^6^

According to a local study, among 99 subjects with T1DM 16 (16.2%) and 53 (53.5%) were positive for IA2 and GAD-65 respectively, while 10 (10.1%) tested positive for both autoantibodies.^7^ However, there are other antibodies like insulin autoantibodies (IAA) and islet cell antibody (ICA) which have not been looked for in Pakistani children. There are two recent studies discussing that the existence of anti-Glutamic Acid Decarboxylase (anti GAD) with various islet cell antibodies in children is concomitant with leisure advancement of Type-1 diabetes.^8^ Being the largest in province, we get referrals from different regions of the country, hence this study will help us more to understand the autoimmunity pattern of Pakistani children in developing diabetes. Also, future studies among first degree relatives of diabetics can be conducted to identify pre-clinical diabetes, allows for less diabetic keto-acidosis, early initiation of insulin therapy, and the potential to delay or prevent diabetes onset. With the advent of safe and specific therapies to block disease specific immune cells our study would provide major ground to use the new drugs in prolonging the disease onset. Moreover, this study opens doors for the detection of other organ specific antibodies like thyroid as the prevalence of thyroid antibodies among anti-GAD positive patients is almost twice as high as among anti-GAD negative.^9^

The rationale of our study is to evaluate the pattern of antibodies in our population of diabetic children so to expidite targeting for prevention and recent intervention in pre diabetics of our country. Moreover, the preservation of C-peptide secretion prevents or delays diabetes related complications^10^. so, its level will help us to identify the population more at risk of developing T1DM related complications like vasculo-pathy and nephropathy.

## METHODS

This cross-sectional study was conducted at Department of Pediatric endocrinology, National Institute of Child Health, Karachi during August 2019 to February 2020. Children from age two years to fifteen years having T1DM for more than a month period were enrolled in the study. There were 98 patients fulfilling the inclusion criteria.

### Ethical Approval

After approval from IERB (Ex-01/2021; dated February 16^th^, 2021) informed written consent was taken before enrolment.

Immuno specific insulin and C-peptide quantitative sandwich enzyme immunoassay Kits (Cat# E29-072& E29-071, USA) were used to estimate C- peptide in serum. Human islet cell antibody (ICA), insulin auto antibodies (IAA), glutamic acid decarboxylase (anti GAD) and Insulinoma associated antigen (IA2) were valued using ELISA. Demographical information, clinical and biochemical information were noted by the principal investigator on a pre-designed proforma. SPSS version 21 was used for data analysis. Frequencies and percentages were computed for qualitative variables like gender, family history, Anti GAD, islet cell antibody, insulin autoantibody (positive/negative). Quantitative variables were presented as mean ±standard deviation for variables like age, duration of Type-1 DM, level of C-peptide, antibody levels. Effect modifiers like age, gender, family history, and duration of T1D were controlled through stratification. Chi-square test and fishers exact test were used where applicable, *p*-value ≤0.05 was considered as significant.

## RESULTS

Out of 98 type-1 diabetic patients 47 (48%) were female and 51 (52%) were male. Although, not statistically significant level of C-peptide was low in around two third of our study group (Table-I) and has a significant reciprocal relationship with duration of diabetes (*p*-0.006) (duration: minimum > one month to maximum 10 years after diagnosis). Age at the time of diabetes has no significant impact on C-peptide level. (Table-I). Anti- GAD was the most frequent antibody found in our subjects (48%), followed by IA2(35.7%) and insulin autoantibodies (7.1%). The whole cohort was negative for islet cell antibody (ICA). (Table-II). Only IAA showed significant association with age (p=0.046). Other effect modifiers, like gender and duration of diabetes were not significantly associated with antibodies positivity.

**Table-I:** Patient characteristics and association of c-peptide level.

Study Outcome		Frequency		Percent
C-Peptide	< 0.8	77(78.3%)		
	≥0.8 (Normal)	21		21.4
Total		98		100
		C-peptide		P-value
< 0.8	≥0.8 (Normal)		
Gender	Female	34(72.3%)	13 (27.7%)	0.149
	Male	43( 84.3%)	8 (15.7%)
Duration of Type-1 DM	< 5years	57 (73.1%)	21 (26.9%)	0.006*
	≥ 5 years	20 (100.0%)	0(0.0%)
Age groups (in years)	< 5 years	19 (76.0%)	6 (24.0%)	0.717
	≥ 5 years	58(79.5%)	15(20.5%)

**Table-II:** Level of antibodies against islet cells in Type-1 Diabetic Pakistani children.

Study Outcome		Frequency (%)
Anti-GAD	Negative	51 (52%)
	Positive	47 (48%)
Total		98 (100%)
Insulinoma- associated antigen 2(IA2)	Negative	63 (64.3%)
	Positive	35 (35.7%)
Insulin auto antibodies	Negative	91 (92.9%)
	Positive	7 (7.1%)
Islet Cell antibody	Negative	98 (100%)
	Positive	0 (0%)

Total		98 (100%)

## DISCUSSION

We evaluated the frequency of different Type-1 diabetes related autoantibodies and their relationship with the age at onset and duration of diabetes in children. At least one antibody was found positive in a round 90% of the children affected with Type-1 DM. Data on β-cell antibodies from Qatar showed 75.5% positivity^11^ and from Iran reported to be 80% at first presentation, which is then declined as the beta cells of the pancreas decrease in number.^12^

A multicenter study from China reported to have 61.01% of their T1DM children positive for beta cell antibodies at initial diagnosis. However, they only checked for anti-GAD, IAA, and ICA.^13^ Studies from India proved that antibody positivity reaches from 55% when only Anti GAD and IA2 were checked, while it reached up to 96% when all five antibodies against Type-1 diabetes were estimated.^9^ The reported prevalence of idiopathic Type-1 diabetes (iAb-) is high (30%) in one study^14^ but remains low (2.2%) only if all five antibodies are estimated.^14^,^15^

Among 98 Type-1 diabetics 47 were females and 51 were males similar to a local study. Mean age at diagnosis was 11.0 ± 5.2 years (range 1.6-21.7 years) in the mentioned study, that is also similar to our data where two third of the children were above five years of age.^7^ Our data represented that antibodies in T1 diabetic children are more likely to be positive if checked within five years, than after five years of establishing a diagnosis. This is similar to other studies where median duration of diabetes was 34.0 (12.7-69.7) months.^12^ Another report also showed significant positive antibodies titer within 11 months of diagnosing diabetes.

We found Anti-GAD antibody among most of diabetic children and adolescents followed by Insulinoma- associated antigen 2(IA2 and insulin autoantibodies (IAA). This is similar to available local data where 53 (53.5%) were GAD-65 positive and 16 (16.2%) were IA2 positive.^7^ one international studies also supports same findings where anti-GAD and anti-IA2 auto-antibodies were found in 76.92% and 62.82% respectively in a mix group of newly and established diabetic children.^16^ Hence, these two antibodies can detect patients early and can persist several years after diagnosis of T1D. This is also alike to the global data where IA2 found to be the most frequent antibody in T1DM.^17^

Our data represents IAA to be detected in 7(7.1%) in contrast to positivity for this antibody from India (63%) 9(Qatar (40.08%)11 and Iran (21.8%)^12^ respectively. This may be due to differences in genetic patterns, age of onset and duration of diabetes.

Our cohort was negative for islet cell antibodies, this is in contrast to the data from a nearby country where ICA was the most commonly found antibody in 66.2%, followed by anti-GAD (56.3%), IA-2A (40.1%) in 142 subjects with different ages and duration of diabetes.^12^ Another study from Qatar reported ICA to be positive in 53.4% of T1DM.^11^

Recent Meta analyses states that the number of iAb determined is more predictive of development of Type-1 diabetes rather than the specific iAb profile.^13^,^17^ Though, we did not look for znt8 antibodies but international studies support its presence to be (40-60%) in newly diagnosed diabetics. However, a study from India shows only 20.08% positive znt8 antibodies. Mean duration of 6.3± 4 years after diagnosis of diabetes could be an explanation to this difference.^9^ Hence the author recommends complete antibody profile including znt8 antibody.

We estimated c-peptide levels in our targeted population and did not find significant differences in relation to gender and age groups. However, its levels significantly declined with the increasing duration of diabetes. This is similar to a recent local study in which C-peptide levels were decreased with longer duration of T1DM but not statistically significant.^7^ Our results are supported by Leighton E et al where C-peptide has been shown to correlate with longevity of disease.^6^ Few other studies from the world also emphasize on stabilization of c-peptide levels more than five years after diagnosis.^18^,^19^

### Limitations

Due to financial constraints; we could not perform the C -peptide level at the time of diagnosis and then follow it to see the pattern of its decline. Further, znt8 antibody could not be assessed which affects the overall positivity of the T1DM antibodies in children as per literature. Despite that, this is probably the first study conducted here with more than two antibodies detection. Previously published local study did not incorporate IAA and ICA in our population. Hence, the authors recommend complete antibodies profile along with c-peptide value when T1DM is diagnosed, as well as on long term follow-up for better understanding of changes in immune function and stronger evidence of beta cells preservice.

## CONCLUSION

Diabetic children had high levels of anti-GAD antibodies, insulin autoantibodies and anti (IA2) but islet cells antibodies were negligible in our population when checked at a point of time. C-peptide may be found normal in a portion of diabetic children but its level declines with increasing duration of diabetes and this fall is steeper in younger children.

### Recommendation

There is other disease associated antibodies which need to be performed like antibodies thyroid gland and celiac antibodies which we could not perform to avoid financial burden on the family. Other than that, we recommend detection of antibodies in non-diabetic siblings of patients as well.

### Authors’ Contribution:

**MR** designed and did statistical analysis & editing of manuscript, is responsible for integrity of research.

**MA** conceived designed and helped in data collection.

**ZAK** did data collection and manuscript writing.

**MNI** did review and final approval of manuscript.
